# GTPase of the Immune-Associated Nucleotide Protein 5 Regulates the Lysosomal Calcium Compartment in T Lymphocytes

**DOI:** 10.3389/fimmu.2017.00094

**Published:** 2017-02-07

**Authors:** Daniel Serrano, Farnaz Ghobadi, Guylain Boulay, Subburaj Ilangumaran, Christine Lavoie, Sheela Ramanathan

**Affiliations:** ^1^Immunology Division, Department of Pediatrics, Université de Sherbrooke, Sherbrooke, QC, Canada; ^2^Department of Pharmacology-Physiology, Faculty of Medicine and Health Sciences, Université de Sherbrooke, Sherbrooke, QC, Canada; ^3^Centre de recherche clinique, Université de Sherbrooke, Sherbrooke, QC, Canada

**Keywords:** GIMAP5, calcium, T cells, lysosomes, lyp mutation

## Abstract

T lymphocytes from *Gimap5^lyp/lyp^* rats carrying a recessive mutation in the GTPase of immune-associated protein 5 (*Gimap5*) gene undergo spontaneous apoptosis. Molecular mechanisms underlying this survival defect are not yet clear. We have shown that *Gimap5^lyp/lyp^* T lymphocytes display reduced calcium influx following T cell antigen receptor (TCR) stimulation that was associated with impaired buffering of calcium by mitochondria. Here, we investigated the subcellular localization of GIMAP5 and its influence on Ca^2+^ response in HEK293T cells and T lymphocytes. The more abundantly expressed GIMAP5v2 localizes to the lysosome and certain endosomal vesicles. *Gimap5^lyp/lyp^* T lymphocytes showed increased accumulation of calcium in the lysosomes as evidenced by Gly-Phe β-naphthylamide (GPN) triggered Ca^2+^ release. As a corollary, GPN-induced Ca^2+^ flux was decreased in HEK293T cells expressing GIMAP5v2. Strikingly, TCR stimulation of rat, mouse, and human T lymphocytes increased lysosomal calcium content. Overall, our findings show that lysosomes modulate cellular Ca^2+^ response during T cell activation and that GIMAP5 regulates the lysosomal Ca^2+^ compartment in T lymphocytes.

## Introduction

In T lymphocytes, calcium influx induced by T cell antigen receptor (TCR) stimulation modulates up to 75% of genes implicated in survival and proliferation through activation of calcineurin and NFAT, leading to IL-2 gene expression (1). TCR engagement triggers the activation of LCK and ZAP70 tyrosine kinases, which phosphorylate many substrates including the scaffolding protein Linker for Activation of T cells, leading to the assembly of a multimolecular signaling platform at the plasma membrane (2). Phospholipase Cγ (PLCγ) that is recruited to this complex becomes activated and hydrolyzes the membrane-bound phosphatidylinositol 4,5 bisphosphate (PIP_2_) to generate inositol 1,4,5-trisphosphate (IP_3_) and diacylglycerol (DAG). IP_3_ binds to its receptor IP_3_R on endoplasmic reticulum (ER) and triggers Ca^2+^ release from the ER store, resulting in a conformational change in the ER-localized STIM1 protein (3, 4). This event relays a signal to open the Ca^2+^ release-activated Ca^2+^ (CRAC) channel on the plasma membrane, inducing capacitative Ca^2+^ entry from the extracellular milieu (5, 6). CRAC channels are the major store-operated channels (SOC) in T lymphocytes (7, 8). TCR stimulation by antigen induces sustained Ca^2+^ influx via CRAC channels leading to T cell proliferation (9). Following sustained Ca^2+^ entry via SOC channels on the plasma membrane, the rising concentration of cytosolic Ca^2+^ ([Ca^2+^]_c_) activates the Ca^2+^ uniporter on the mitochondrial membrane that induces a slow, membrane potential-driven uptake of Ca^2+^, which is released later via the Na^+^/Ca^2+^ exchanger (10). This Ca^2+^ uptake by mitochondria ([Ca^2+^]_m_) is necessary to prevent feedback inhibition of the SOC channel activity by the raising cytosolic Ca^2+^ concentration (11, 12).

In the BB-DP strain of rats, homozygosity for the *lyp* mutation causes a 5- to 10-fold reduction in CD4^+^ T lymphocyte numbers and absence of CD8^+^ T lymphocytes in secondary lymphoid organs (13–16). Mature T cells in these rats undergo spontaneous apoptosis soon after they emigrate from the thymus and enter peripheral circulation. The half-life of recently emigrated mature T cells is markedly reduced in BB-DP rats compared to non-lymphopenic rats (3 versus >15 days) (16–20). The *lyp* allele arises from a frameshift mutation within the GTPase domain of the immune-associated nucleotide-binding protein 5 (*Gimap5*) gene, resulting in a hypothetical protein lacking 223 amino acids at the C-terminus that is not expressed (21, 22). GIMAP5 is a member of the GIMAP family that is implicated in immune functions in plants and mammals, and arose by convergent evolution (23, 24). In mice, GIMAP5 was shown to interact with members of the Bcl-2 family of antiapoptotic proteins (25–28), whereas another study implicated GIMAP5 in ER stress response (29). In contrast, we have shown that in rat T cells, endogenous GIMAP5 resides in a cellular compartment distinct from mitochondria and ER and that neither endogenous rat GIMAP5 nor the overexpressed protein interacts with Bcl-2 (30). Yet, loss of GIMAP5 impairs mitochondrial membrane integrity (31). Despite a decade of efforts by several groups, there is a lack of consensus on the mechanisms through which GIMAP5 promotes the survival of quiescent T lymphocytes.

We have shown previously that signals through TCR and IL-7 receptor that are required for the survival of T lymphocytes in the secondary lymphoid organs (32–36) were reduced in the absence of functional Gimap5 (37, 38). Furthermore, we showed that T lymphocytes from *Gimap5^lyp/lyp^* rats display defective Ca^2+^ flux in response to TCR signaling (39). However, the mechanisms by which GIMAP5 regulates cellular Ca^2+^ homeostasis are not yet clear. We observed that the loss of *Gimap5* did not influence Ca^2+^ release from the ER in primary T lymphocytes (39). On the other hand, GIMAP5 deficiency in T lymphocytes compromised the ability of the mitochondria to sequester Ca^2+^ that enters via SOC channels (40). Consistent with this, overexpression of GIMAP5 in HEK293T cells resulted in increased Ca^2+^ accumulation within the mitochondria (40). Given that GIMAP5 is not physically located on the mitochondria (30), how GIMAP5 regulates mitochondrial Ca^2+^ is not known. Here, we show that GIMAP5 regulates lysosomal Ca^2+^ and that lysosomes contribute to Ca^2+^ homeostasis during T cell activation.

## Results

### GIMAP5 Is Localized on Lysosomes and Certain Vesicles through the C-Terminal Anchor

**Figure 1:**
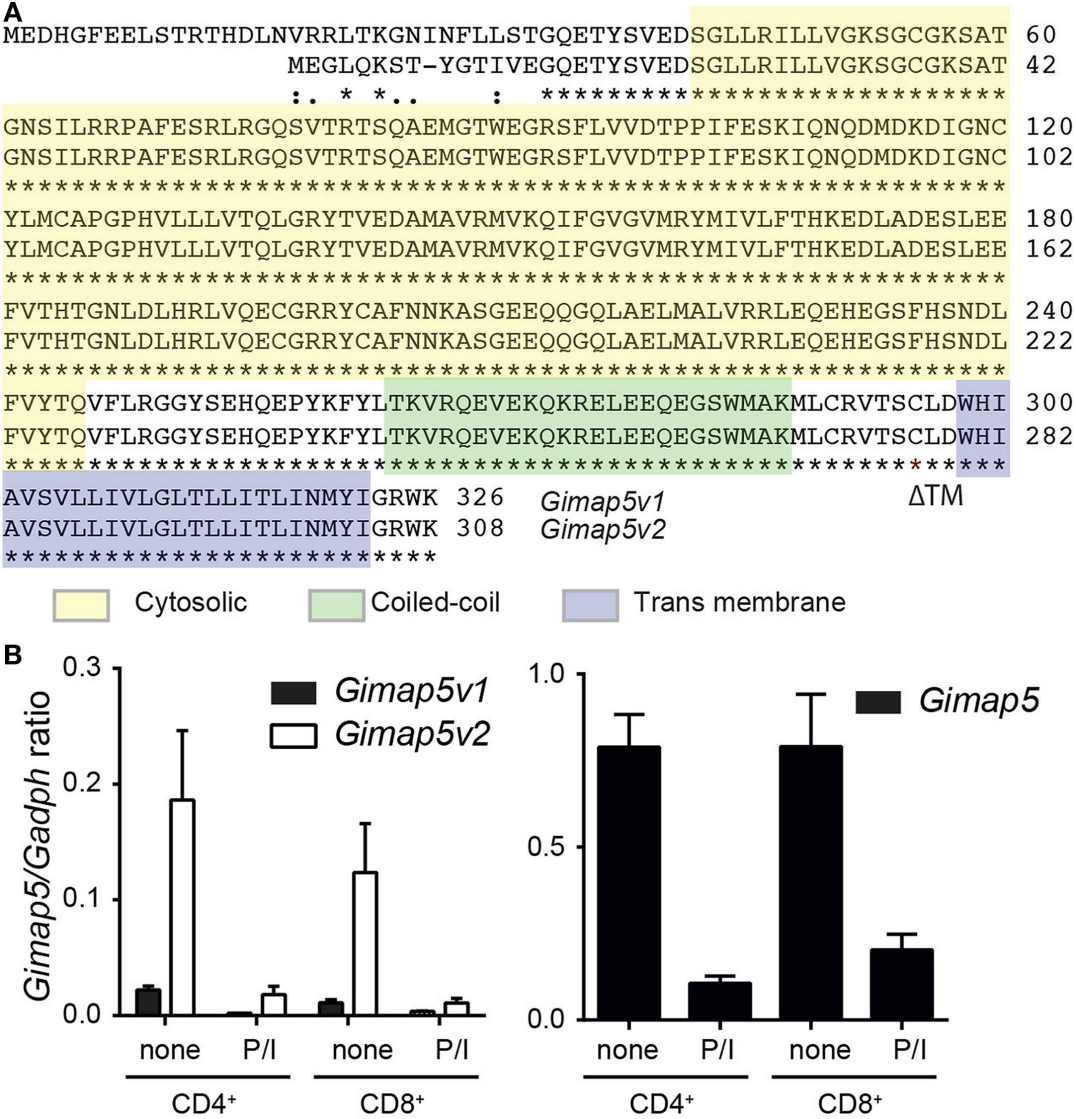
**Expression of *Gimap5* isoforms in rat T lymphocytes**. **(A)** Sequence alignment of v1 and v2 variants of rat GIMAP5 protein. The cytosolic (yellow), coiled-coil (green) transmembrane (transmembrane; blue) domains are indicated by a box. The predicted structure was obtained from http://www.uniprot.org/uniprot/Q0R3W7. **(B)** *Gimap5* mRNA expression in primary T lymphocytes was measured by RT-qPCR. CD4^+^ and CD8^+^ T lymphocytes were purified from lymph nodes of *Gimap5^lyp/lyp^* and control rats and stimulated with PMA/ionomycin (P/I) for 24 h. Expression of the *Gimap5* isoforms v1 and v2 (left panel) and total *Gimap5* (right panel) in control and stimulated lymphocytes was measured by RT-qPCR using specific primers.

**Figure 2:**
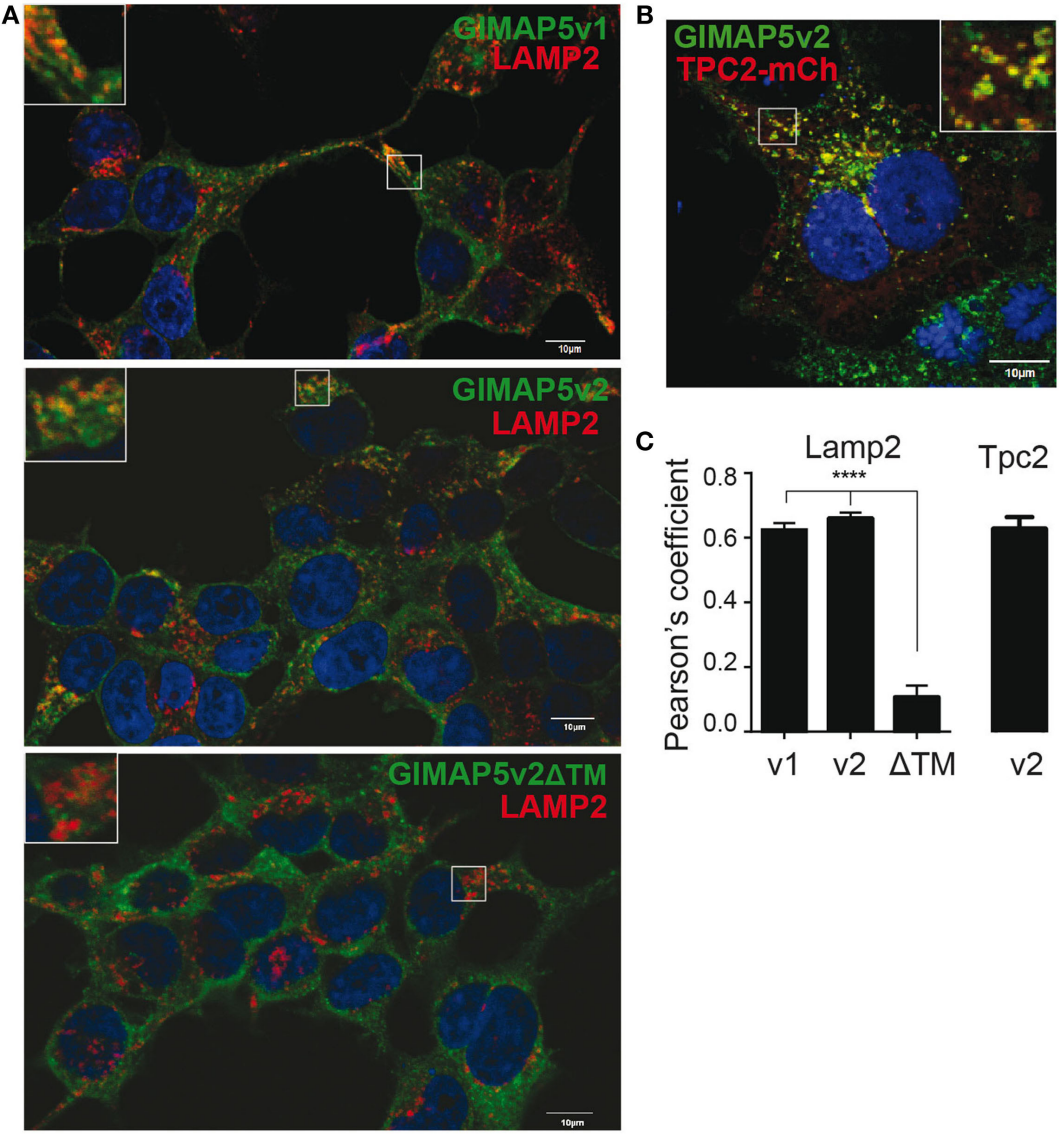
**GIMAP5 variants are anchored on the lysosomal membrane through C-terminal transmembrane domain**. **(A–C)** Stable transfectants of HEK293T cells expressing full-length or C-terminal transmembrane domain-deleted GIMAP5 constructs tagged with EGFP were labeled with **(A)** anti-LAMP2 antibody **(B)** or cherry-TPC2 and analyzed by confocal microscopy. Bar represents 10 µm. Insets show the area analyzed for colocalization. **(C)** Colocalization values are expressed as Pearson’s coefficient. Representative data from four experiments with 6–14 cells analyzed per experiment **(A)** and three experiments with 2–5 cells analyzed per experiment **(B)** are shown.

**Figure 3:**
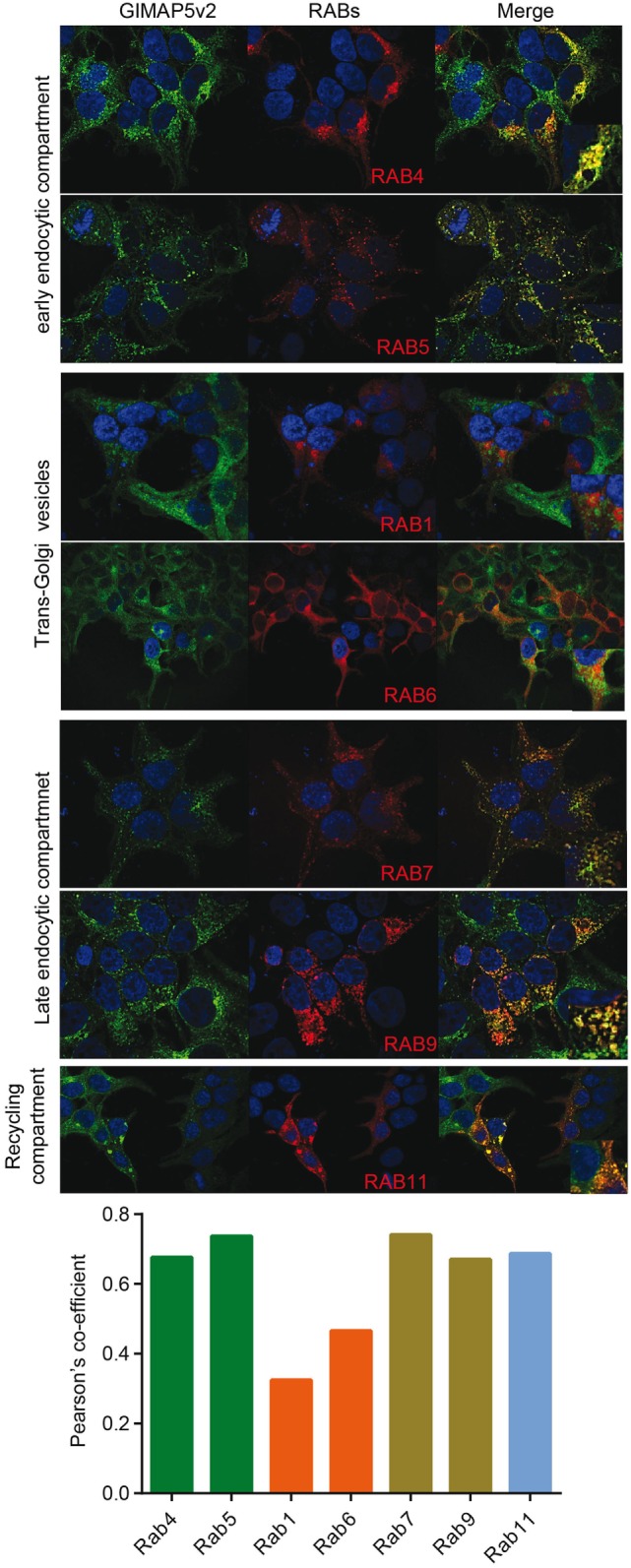
**GIMAP5v2 is expressed on certain vesicles**. 293T cells expressing EGFP-tagged GIMAP5v2 were transiently transfected with plasmids expressing pmRFP-Rab5, pmRFP-Rab7, pDSRed-Rab9, pDSRed-Rab11, HA-Rab1a, HA-Rab4, or HA-Rab6a. HA-tagged Rabs were labeled with anti-HA antibody followed by secondary anti-mouse antibody tagged with Alexa Fluor 633 and analyzed by confocal microscopy. Colocalization values are expressed as Pearson’s coefficient. Representative data from two experiments with four to six cells analyzed per experiment are shown.

Rat T lymphocytes express two isoforms of GIMAP5 that differ in the N-terminal avrRpt2-induced gene 1 (AIG) domain, but possess a coiled-coil domain and a transmembrane (TM) domain (Figure 1A). Expression of the shorter variant (GIMAP5v2) is 8- to 10-fold higher in comparison to the longer variant (GIMAP5v1) in primary rat T lymphocytes (Figure 1B, left panel). However, following activation of T lymphocytes, the expression of both isoforms was reduced by 24 h (Figure 1B) and corresponds to the previous observation that *in vivo* antigen stimulation temporarily overcomes the requirement for GIMAP5 (19). The TM domain, which is common to both GIMAP5 variants, targets the protein to certain intracellular membranes. Human and murine GIMAP5 proteins have been reported to be present on lysosomes in T lymphocytes (41). We constructed full-length and TM-deletion constructs of GIMAP5v1 and GIMAP5v2 to evaluate their subcellular distribution and functions (Figure S1 in Supplementary Material). We observed that overexpressed rat GIMAP5 variants colocalized with lysosomal membrane proteins such as LAMP2 and TPC2 (Figure 2), but not with Lysotracker Red that predominantly localizes to the lysosomal lumen (Figure S2 in Supplementary Material). Deletion of TM domain in GIMAP5v2ΔTM abolished its lysosomal localization (Figure 2A), suggesting that GIMAP5 is anchored on the lysosomes through the C-terminal TM domain. Accordingly, real-time monitoring of lysosomes in HEK293T or HeLa cells transfected with GIMAP5v2 showed a vesicular pattern of staining; some of these vesicles also stained for LAMP1 and were in constant movement in transfected HEK293T cells (Video S1 in Supplementary Material). In addition to lysosomes, GIMAP5 also colocalized with Rabs 4, 5, 7, 9, and 11, which are markers of early- and late-endocytic and recycling compartments but not with trans-Golgi vesicles (Figure 3).

We have shown previously that overexpressed GIMAP5v2 colocalized with microtubules (40). To determine whether this association resulted from the interaction of lysosomes with microtubules, we expressed cherry-α-tubulin in HEK293T cells stably expressing FLAG-tagged GIMAP5 variants. As shown in Figure S4A in Supplementary Material, both variants colocalized with microtubules, with a significantly higher co-localization coefficient for GIMAP5v2. On the other hand, both the variants of GIMAP5 did not colocalize with actin (Figure S3B in Supplementary Material). Deletion of the TM domain resulted in a diffuse cytoplasmic staining and showed increased colocalization with microtubules, probably due to the loss of the restraints posed by the TM anchor. Accordingly, the expression of GIMAP5v2ΔTM did not show any vesicular pattern of staining when compared to the full-length protein (Video S2 in Supplementary Material).

**Figure 4:**
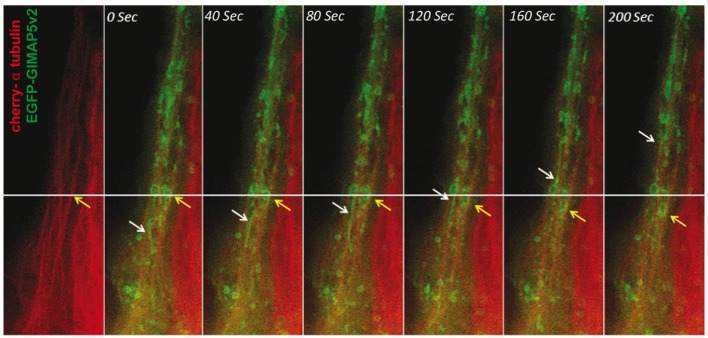
**GIMAP5v2-expressing vesicles move along microtubules**. HeLa cells were transfected with vectors containing cherry-α tubulin and EGFP-tagged GIMAP5v2. White and yellow arrows track the movement of certain vesicles. The vesicles are either filamentous or spherical. The speed of their movement is not uniform. Bar represents 10 µm. Frames were taken from Video S3 in Supplementary Material.

The above results indicated that GIMAP5 is inserted in the lysosomal and vesicular membranes through the C-terminal anchor and suggested that the N-terminal region of GIMAP5 resides in the cytosol and interacts with microtubules. Distinct motor proteins are involved in the movement of organelles on microtubules (42, 43). Because of the possible implication of GIMAP5 in organelle movement, we investigated its colocalization with the molecular motor proteins, kinesin and dynein, that interact with microtubules (42). Significant colocalization of rGIMAP5v2 occurred with kinesin but not with dynein, suggesting that GIMAP5 may be involved in kinesin-mediated retrograde cargo movement (Figures S4 and S5 in Supplementary Material). To visualize the movement of GIMAP5-containing vesicles, HEK293T cells that stably express EGFP-tagged GIMAP5v2 were transfected with cherry-tagged α-tubulin and the dynamics of GIMAP5v2-containing vesicles was assessed by epifluorescence microscopy. Microtubule-associated GIMAP5v2-containing vesicles showed directional movement along the microtubules (Figure 4; Video S3 in Supplementary Material).

### GIMAP5v2, but Not GIMAP5v1, Regulates Cellular Ca^2+^

**Figure 5:**
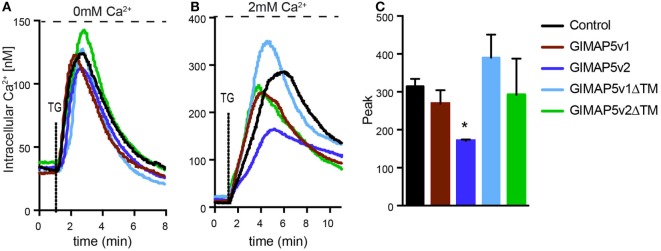
**Full-length GIMAP5v2 regulates intracellular Ca^2+^ homeostasis**. Cytosolic Ca^2+^ concentration following the addition of TG was measured using Fura-2 in HEK293T stable cell lines expressing empty vector or different GIMAP5 constructs following the addition of TG. **(A)** Ca^2+^ release from the endoplasmic reticulum (ER) was measured in medium containing 0 mM Ca^2+^ and 0.5 mM EGTA following addition of TG. **(B)** Increase in cytosolic Ca^2+^ induced by release of the ER store and influx from the extracellular medium was measured following the addition of TG in the presence of 2 mM Ca^2+^. For all experiments, cells were plated on cover slips 48 h before analysis. Each experiment is an average of 40–60 cells. Representative data from three to five independent experiments with comparable results are shown. **(C)** Peak value for cytosolic Ca^2+^ influx shown in **(B)** is plotted as a bar graph and compared by Student’s *t*-test (*p < 0.05).

While the absence of GIMAP5 results in precocious death of T lymphocytes, its overexpression also induces apoptosis in T cell lines (29). In addition to apoptosis, T lymphocytes from *Gimap5^lyp/lyp^* rats showed diminished Ca^2+^ influx from the extracellular medium following activation through the T cell receptor (39). As *Gimap5v2* was more abundant than *Giamp5v1* in rat T lymphocytes (Figure 1B), it is possible that GIMAP5v2 mediates the regulation of Ca^2+^ homeostasis. To determine whether *Gimap5v1* also regulates cellular Ca^2+^, we studied the Ca^2+^ flux in HEK293T cells expressing GIMAP5v1. We have shown previously that expression of GIMAP5v2 in HEK293T cells did not induce apoptosis, but modulates Ca^2+^ flux in a manner reminiscent of the responses observed in T lymphocytes (30, 39, 40). In the absence of extracellular Ca^2+^, thapsigargin (TG)-induced release of Ca^2+^ from the ER was comparable in cells expressing GIMAP5v1, GIMAP5v2, or the ΔTM constructs (Figure 5A), suggesting that GIMAP5 does not influence Ca^2+^ release from the ER store. These observations are also in agreement with our previous report that Ca^2+^ release from the ER was not affected by the *lyp* mutation in rat T lymphocytes (39). On the other hand, TG-induced Ca^2+^ influx in the presence of extracellular Ca^2+^ was significantly reduced in cells expressing GIMAP5v2 but not in cells expressing GIMAP5v1 or GIMAP5v2ΔTM (Figures 5B,C), indicating that the N-terminal domain of GIMAP5v2 and the membrane anchor is required for regulating Ca^2+^ influx.

### GIMAP5v2 Does Not Influence Mitochondrial Ca^2+^ Content

**Figure 6:**
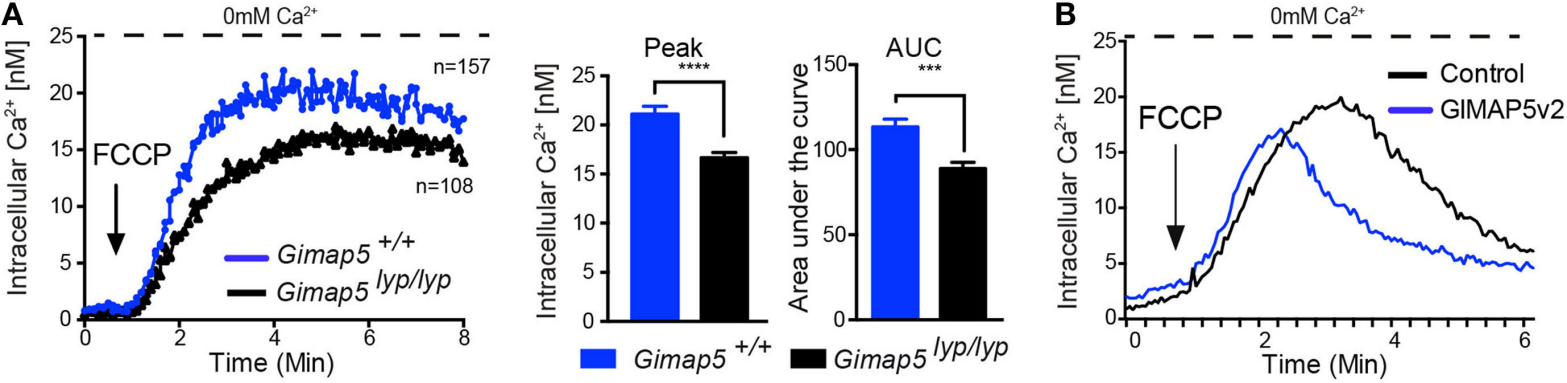
**Influence of GIMAP5v2 on Ca^2+^ release from mitochondria**. Cytosolic Ca^2+^ concentration following the addition of FCCP (50 µM) was measured in the absence of extracellular Ca^2+^ in Fura-2-loaded **(A)** CD4^+^ T lymphocytes purified from *Gimap5^lyp/lyp^* and control rats and in **(B)** Fura-2-loaded HEK293T stable cell lines expressing empty vector or GIMAP5v2. For all experiments, cells were plated on cover slips 48 h before analysis. Each experiment is an average of 40–60 cells. Representative data from two independent experiments are shown. Peak value for cytosolic Ca^2+^ influx for primary T lymphocytes is plotted as a bar graph and compared by Student’s *t*-test (*p < 0.05).

Even though T lymphocytes from *Gimap5^lyp/lyp^* rats display impaired mitochondrial Ca^2+^ uptake, this defect was not observed when the lymphocytes were permeabilized (40), suggesting that the primary defect in cellular Ca^2+^ homeostasis in these lymphocytes may not be at the level of mitochondria. To determine whether there were differences in the mitochondrial Ca^2+^ content in resting state, we probed the mitochondrial content by releasing its Ca^2+^ using Carbonyl cyanide 4-(trifluoromethoxy)phenylhydrazone (FCCP), which uncouples the mitochondrial membrane potential. As shown in Figure 6A, the amount of Ca^2+^ released from the mitochondria in *Gimap5^lyp/lyp^* T lymphocytes was slightly lower than that observed in T lymphocytes from control rats. These observations suggest that the mitochondrial Ca^2+^ content may be reduced in T lymphocytes due to the *lyp* mutation. It is also possible that this reduction in the mitochondrial Ca^2+^ content is a refection of the modest loss in mitochondrial membrane potential *ex vivo* (30). To determine whether GIMAP5 intrinsically influences the mitochondrial Ca^2+^ stores directly, we assessed cytosolic Ca^2+^ released by FCCP in control and GIMAP5v2-expressing HEK293T cells. In the absence of extracellular Ca^2+^, the release of Ca^2+^ from mitochondria by FCCP was comparable between control and GIMAP5v2-expressing cells (Figure 6B). These observations suggest that GIMAP5 minimally influences the Ca^2+^ content of mitochondria at steady state. However, similar to the results obtained with TG, the entry of Ca^2+^ from extracellular milieu following FCCP was still significantly decreased in HEK293T cells expressing GIMAP5v2 (40). Thus, even if the Ca^2+^buffering capacity of mitochondria is higher in GIMAP5v2-expressing 293T cells as shown by us previously (40), the differences observed in Ca^2+^ influx from extracellular medium cannot be explained by alterations in the mitochondrial Ca^2+^ content in the steady state.

### GPN-Induced Ca^2+^ Release Is Increased in the Absence of GIMAP5v2

**Figure 7:**
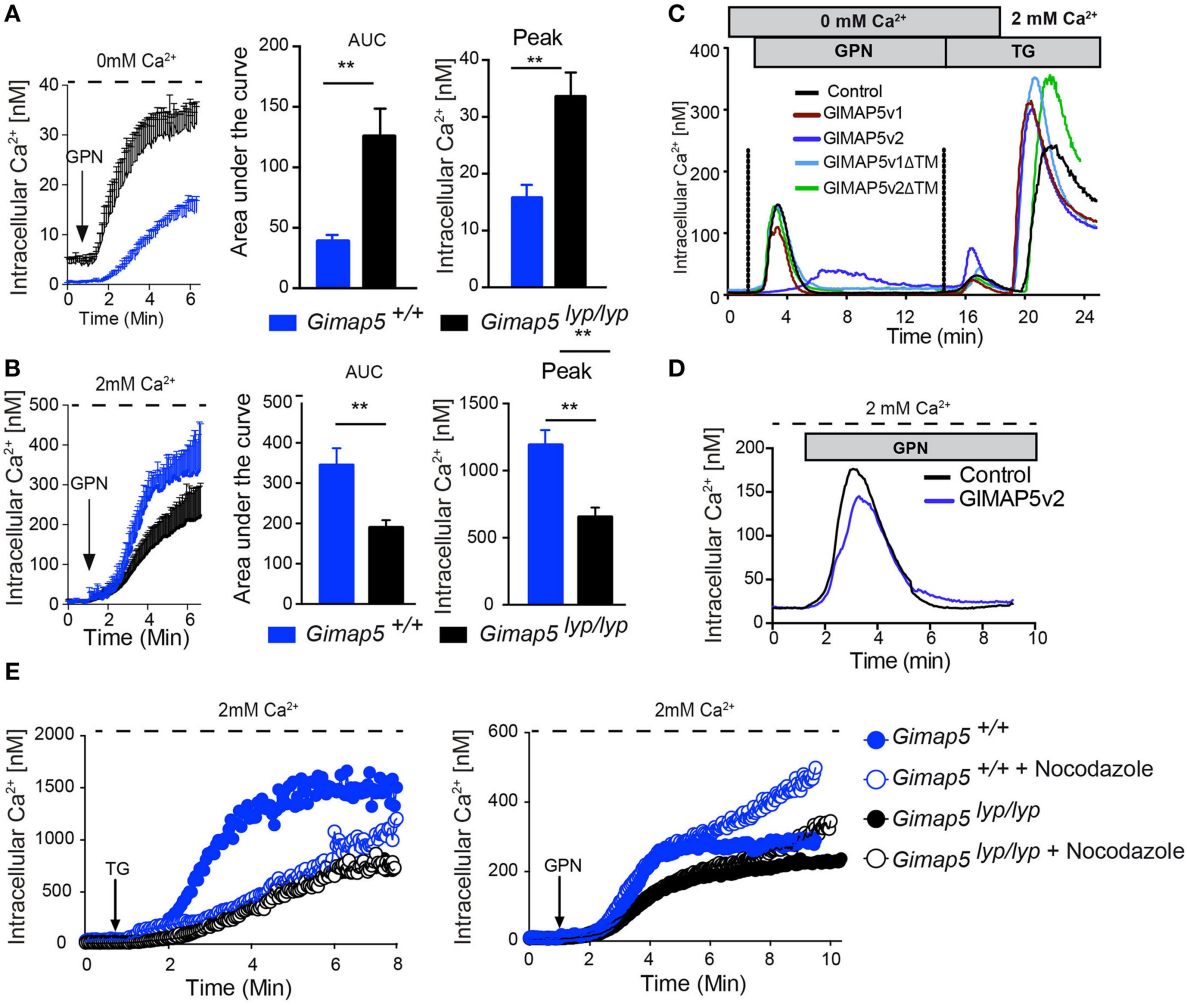
**GPN-mediated release of Ca^2+^ is increased in the absence of GIMAP5**. Peripheral CD4^+^ T lymphocytes purified from *Gimap5^lyp/lyp^* and control rats were loaded with Fura-2 and intracellular Ca^2+^ was measured as described. The arrow indicates the time of addition of GPN (100 µM). Cytosolic Ca^2+^ was measured following addition of GPN in the absence **(A)** or presence **(B)** of extracellular Ca^2+^. The histograms represent the area under curve (AUC) and the peak values. Student’s *t*-test (**p < 0.005). **(C)** Cytosolic Ca^2+^ was measured using Fura-2-labeled stable transfectants of the indicated GIMAP5 constructs. The cells were maintained in Ca^2+^-free medium (0 mM Ca^2+^ and 0.5 mM EGTA), and GPN and TG were added sequentially as indicated before adding Ca^2+^ to the extracellular medium. **(D)** Ca^2+^ release from stable transfectants of the vector control or GIMAP5v2 following addition of GPN in the presence of extracellular Ca^2+^. **(E)** Cytosolic Ca^2+^ was measured following addition of TG or GPN in the presence of extracellular Ca^2+^ in T lymphocytes pretreated or not with nocodazole (2 µM) for 30 min.

As GIMAP5v2 localizes to the lysosomal membrane (Figure 2), interacts with kinesin (Figure S4 in Supplementary Material), and the GIMAP5v2-containing vesicles (Figure 4) move along the microtubule network, it is possible that GIMAP5v2 might be involved in the regulation of lysosomal Ca^2+^ homeostasis. We assessed the possibility that GIMAP5 influences lysosomal Ca^2+^ content. Hydrolysis of Gly-Phe β-naphthylamide (GPN) within the lysosomes by cathepsin C results in osmotic lysis of the acidic compartment (44). Thus, GPN-mediated release is a reflection of the Ca^2+^ content of lysosomes. Treatment with GPN resulted in a higher cytosolic Ca^2+^ content in T lymphocytes from *Gimap5^lyp/lyp^* rats when compared to T lymphocytes from control rats (Figure 7A). When GPN was added in the presence of extracellular Ca^2+^, influx from extracellular milieu was observed in T lymphocytes suggesting that emptying of lysosomal stores can induce Ca^2+^ influx through the plasma membrane in T lymphocytes (Figure 7B). In support, acidic stores have been shown to express STIM1 and STIM2 in human platelets (45). Strikingly, *Gimap5^lyp/lyp^* T lymphocytes, despite showing increased lysosomal Ca^2+^ release (Figure 7A), displayed substantially reduced Ca^2+^ influx from the extracellular medium when compared to controls (Figure 7B).

To understand the mechanisms by which GIMAP5 influences lysosomal Ca^2+^ compartment, we probed the lysosomal Ca^2+^ content in HEK293T cells stably expressing different constructs of GIMAP5. The GPN-induced Ca^2+^ release was reduced in cells expressing GIMAP5v2, but not in cells expressing empty vector, GIMAP5v1, or GIMAP5v2ΔTM (Figure 7C). These observations suggest that the presence of full-length GIMAP5v2 is required and is sufficient to prevent the accumulation of Ca^2+^ by lysosomes. To determine whether the lysosomal and ER Ca^2+^ stores are related and to determine whether they influence each other, we sequentially released Ca^2+^ from lysosomes and ER (Figure 7C). The release of Ca^2+^ from the ER after lysosomal release was higher in cells expressing GIMAP5v2. Sequential release of Ca^2+^ from lysosomes and ER in GIMAP5v2-expressing cells resulted in a greater influx of Ca^2+^ from the extracellular milieu when compared to that of control cells. As treatment with TG, FCCP (40), or GPN alone (Figure 7D) resulted in reduced Ca^2+^ influx from extracellular milieu in GIMAP5v2-expressing cells, it suggests that in HEK293T cells, the lysosomal and ER Ca^2+^ stores may be interconnected (46). Due to the fragility of *Gimap5^lyp/lyp^* T lymphocytes, we could not carry out similar experiments in primary T lymphocytes. Nevertheless, our findings on the overexpression system lend support to the idea that dynamic interaction between lysosomes and ER modulates the Ca^2+^ flux response (47).

### Nocodazole Treatment Increases the Ca^2+^ Influx Following GPN Treatment

Mitochondrial movement on cytoskeletal elements to Ca^2+^-rich microdomains is required for the maintenance of the steady influx of the Ca^2+^ (12, 40, 48). In T lymphocytes, soluble mediators such as anti-TCR antibodies or TG requires the movement of mitochondria on microtubules while movement on actin filaments is observed to maintain the Ca^2+^ influx at the immunological synapse (40, 48). Even though the capacity of mitochondria to take up Ca^2+^ is not defective in permeabilized *Gimap5^lyp/lyp^* T lymphocytes, they were not efficient in buffering the Ca^2+^ influx (40). In support, nocodazole treatment of T lymphocytes from control rats resulted in a substantial decrease in TG-induced Ca^2+^ influx through the plasma membrane while minimal effect was observed in T lymphocytes from *Gimap5^lyp/lyp^* rats (Figure 7E, left panel). TG-induced Ca^2+^ influx was decreased as early as 2 min in control T lymphocytes, while no difference was observed in *Gimap5^lyp/lyp^* T lymphocytes (Figure 7E, left panel). These observations complement our previous report that addition of nocodazole prevented mitochondrial Ca^2+^ uptake in T lymphocytes from controls, but not in *Gimap5^lyp/lyp^* T lymphocytes (40). Next we measured Ca^2+^ influx through the plasma membrane following GPN treatment in T lymphocytes that were pretreated with nocodazole. To our surprise, we observed that the lysis of lysosomes and the consequent release of lysosomal Ca^2+^ did not influence Ca^2+^ influx from the extracellular milieu in both control and *Gimap5^lyp/lyp^* T lymphocytes until 6 min (Figure 7E, right panel). At later time points, the Ca^2+^ influx was considerably increased in control T lymphocytes, while the mutant T lymphocytes showed a modest increase. These observations suggest that Ca^2+^ uptake by lysosomes, rather than mitochondrial translocation to Ca^2+^-rich microdomains acts as a brake on sustained Ca^2+^ influx through the plasma membrane and that GIMAP5 may have a role to play in this regulation.

### Lysosomes Accumulate Ca^2+^ Following Crosslinking of TCR

**Figure 8:**
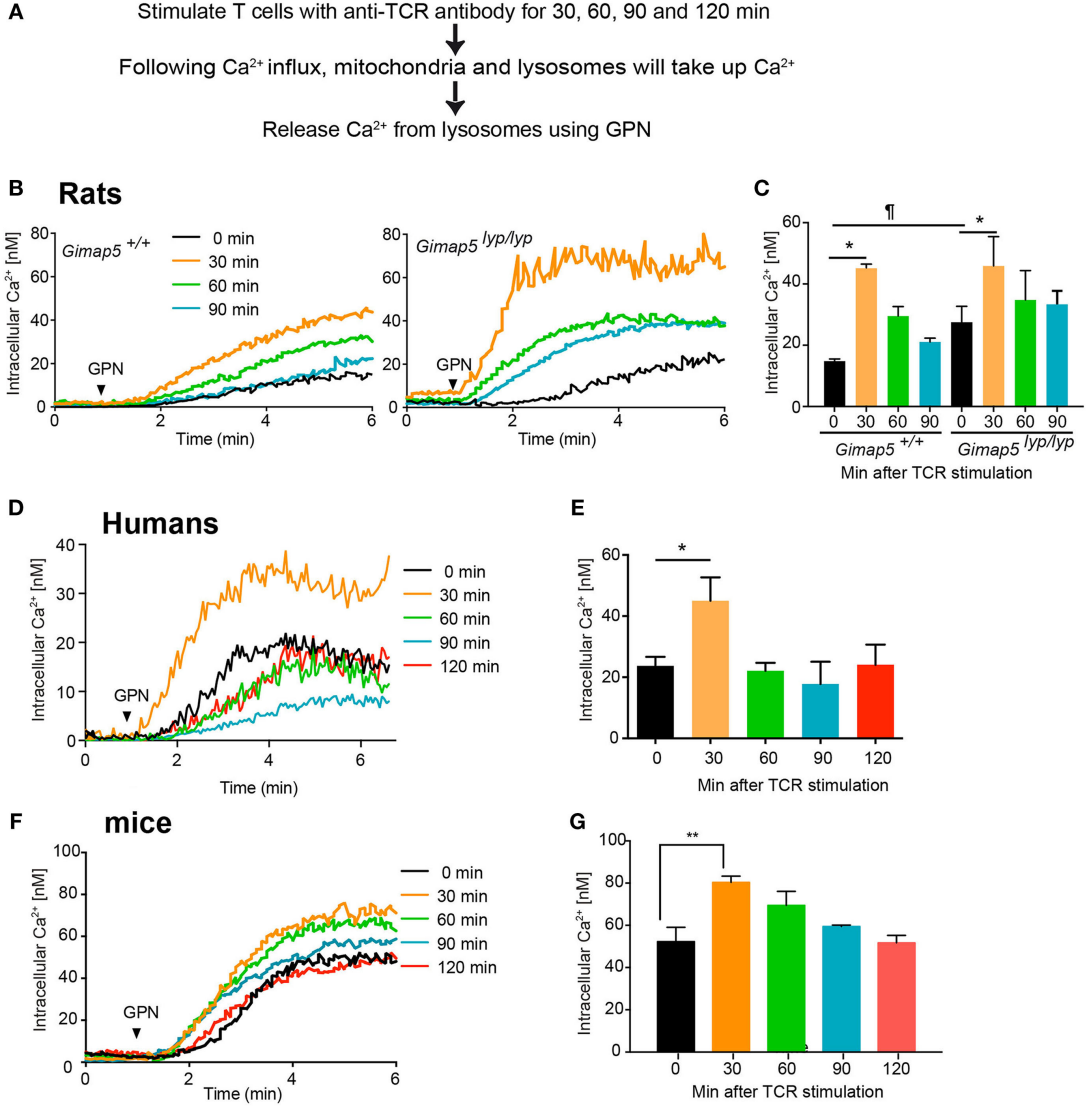
**Lysosomes accumulate Ca^2+^ following activation through T cell receptor**. **(A)** Schematic representation of the experimental setup is indicated. Purified CD4^+^ T lymphocytes from **(B,C)** rats, **(D,E)** humans, and **(F,G)** mice were plated on coverslips and stimulated with anti-TCR antibody (R73 for rats, IP26 for humans, and H57 for mice) for the indicated duration. The lymphocytes were then loaded with Fura-2. Lysosomal Ca^2+^ content was probed with GPN in the absence of extracellular Ca^2+^. Representative data from one experiment are shown in **(B,D,F)**. The average of peak values from three independent experiments is shown in **(C,E,G)**. ^¶^*p* < 0.05 between WT and *Gimap5^lyp/lyp^* unstimulated (*p < 0.05; **p < 0.01).

Given that GIMAP5 appears to regulate lysosomal Ca^2+^ in T lymphocytes, we assessed the involvement of lysosomes in Ca^2+^ homeostasis during T cell activation. Steady influx of Ca^2+^ through the CRAC channel is maintained by the buffering of cytosolic Ca^2+^ by mitochondria (11). Mitochondrial Ca^2+^ increases within 10–15 min following crosslinking of TCR on the cell surface (40, 48). To determine whether lysosomes contributed to the accumulation of Ca^2+^ following T cell activation, we assessed the lysosomal Ca^2+^ content at various time points following TCR stimulation by releasing their Ca^2+^ with GPN. CD4^+^ T lymphocytes from control and *Gimap5^lyp/lyp^* rats were stimulated with anti-TCR antibody R73 in the presence of Ca^2+^. At indicated time points, coverslips containing activated T lymphocytes were transferred to Ca^2+^-free medium and GPN was added to measure the amount of Ca^2+^ released from the lysosomes (Figure 8A). The lysosomal Ca^2+^ content was consistently higher between 30 and 120 min following crosslinking of TCR in T lymphocytes from control and *Gimap5^lyp/lyp^* rats (Figures 8B–C) suggesting that lysosomal Ca^2+^ accumulation may be a late event following TCR activation. Strikingly, *Gimap5^lyp/lyp^* T lymphocytes showed increased TCR-induced lysosomal Ca^2+^ accumulation than control lymphocytes. To determine whether lysosomal Ca^2+^ accumulation following TCR stimulation is a general phenomenon, we carried out similar experiments on human and murine CD4^+^ T lymphocytes. In these lymphocytes, increase in lysosomal Ca^2+^ content occurred at 30 min after TCR stimulation (Figures 8D–G). These observations suggest that (i) lysosomal Ca^2+^ accumulation is a late event during T cell activation and (ii) GIMAP5 prevents lysosomal Ca^2+^ accumulation in the absence of TCR activation.

## Discussion

The data presented here establish a role for GIMAP5 in regulating Ca^2+^ homeostasis by modulating lysosomal Ca^2+^ stores. Lysosomes store significant amounts of Ca^2+^ and contribute to vesicular trafficking, autophagy, endocytosis, and exocytosis in most of the cell types, including immune cells (49). Exocytosis of cytolytic granules in activated cytotoxic T lymphocytes can be triggered by NAADP and/or ADPR, ligands of TPC and TRPM channels present on lysosomes (50). Our findings have revealed a hitherto unknown function for lysosomal Ca^2+^ in T lymphocyte survival and activation and a role for *Gimap5* in regulating this process.

Of the two alternate GIMAP5 transcripts expressed in rat T lymphocytes, the shorter rGIMAP5v2 influences Ca^2+^ homeostasis in a significant manner, while the longer variant had minimal influence on the cellular Ca^2+^ responses. As the two transcripts differ at the N-terminal 32 amino acids, it is likely that the domain structure of rGIMAP5v2 plays an important role in regulating Ca^2+^ homeostasis. This notion is further supported by the higher expression of *Gimap5v2* compared to *Gimap5v1* in T lymphocytes. In accordance with our previous observation that T cell activation transiently rescues *Gimap5-*deficient T lymphocytes from death, *Gimap5* expression is downregulated following T cell activation (19). Two variants of GIMAP5 can be generated in rats and in humans (26, 51), whereas only the GIMAP5v2 is expressed in mice (25). On the other hand, mice possess a functional *Gimap3*, which is a pseudogene in rats and humans (22). One study has shown that loss of *Gimap5* and *Gimap3* is required for the lymphopenic phenotype in mice (52). These findings raise the possibility that *Gimap5v1* in rats and humans, and *Gimap3* in mice may complement or regulate the functions of *Gimap5v2*. Additionally, or alternatively, these GIMAP proteins may form homo- and heterodimers, adding an additional level of complexity to GIMAP5 activity (53).

Our observations confirm and extend the previous report on the lysosomal localization of GIMAP5 (41). GIMAP5 is anchored to the cytosolic side of the lysosomal and vesicular membranes through the C-terminal TM domain, facilitating the cytosolic N-terminal domain to mediate its functions. Even though GIMAP5 is present on endocytic and recycling vesicles that are involved in TCR signaling (54–57), it is not clear at present whether GIMAP5 is implicated in these functions. It is possible that the reduced intensity of tyrosine phosphorylation following activation of *Gimap5* mutant T lymphocytes (37, 38, 40) may be a consequence of defects in specific endosomal compartments. It is also possible that GIMAP5 may have a role in autophagy as GIMAP6, another member of the GIMAP family, has been shown to interact with GABARAPL2, an Atg8 homolog (58).

Current knowledge on the role of lysosomal Ca^2+^ in T cell homeostasis is very limited. Lysosomal Ca^2+^ is implicated in the release of cytotoxic granules (50). Lysosomal storage disorders are associated with immune dysfunctions as a consequence of abnormal accumulation of metabolites (59). However, it is not clear whether Ca^2+^ homeostasis is also affected in T lymphocytes from these patients. Similarly, while mitochondrial dysfunction affects lysosomal activity and autophagy in T lymphocytes, their effects on lysosomal Ca^2+^ have not been studied (60). It is quite probable that deregulation of lysosomal functions are also associated with altered lysosomal Ca^2+^ content. The data presented here suggest that the lysosomal Ca^2+^ content of resting T lymphocytes is maintained at a low level in the presence of GIMAP5 and that it increases during later stages of T cell activation. IP_3_ generated following TCR stimulation empties the ER Ca^2+^ stores and opens the ORAI/CRAC channels on the plasma membrane (7). The incoming Ca^2+^ is buffered by mitochondria that move on microtubules (61). Mitochondria buffer the Ca^2+^ influx during the first 15 min following the opening of CRAC channels induced by the emptying of the ER Ca^2+^ (40, 62). Our observations with lysosomes add additional regulatory steps to this process. The data presented here show for the first time that lysosomal Ca^2+^ content peaks around 30 min following TCR stimulation in rats, mice, and humans (Figure 8). However, we do not rule out the possibility that lysosomes accumulate Ca^2+^ at the same time or immediately after mitochondria. Inhibition of microtubule polymerization prevents mitochondrial movement for Ca^2+^ accumulation and hence the Ca^2+^ influx from the extracellular milieu (12, 40, 48). However, this inhibition of microtubule polymerization does not seem to affect Ca^2+^ influx following GPN-mediated lysis of lysosomes but in fact increases the influx, lends credence to the concept that lysosomes play an important role in regulating Ca^2+^ influx. This increased influx despite the inability of mitochondria to move and buffer this influx suggests that in addition to mitochondria, lysosomal GIMAP5 may be an additional break for the Ca^2+^ influx in T lymphocytes. Thus, lysis of lysosomes removes the restraint imposed by GIMAP5 and/or lysosomal Ca^2+^ and permits increased entry. It is possible that both GIMAP5-mediated restraint (by interacting with cellular components) and lysosomal Ca^2+^ are important as Ca^2+^ influx following GPN and TG addition was higher in HEK293T cells expressing the GIMAP5ΔTM as well as in cells expressing GIMAP5v1 (Figure 7C). Comparison of Ca^2+^ influx observed in Figures 5B and 7C suggests that active ER-lysosomal cross-talk (47) can add additional level of complexity to these interactions (discussed below). It is possible that interaction of GIMAP5 with microtubules prevents the transfer of Ca^2+^ from lysosomes to the ER during ligand-induced ER Ca^2+^ depletion in order to facilitate STIM–ORAI interactions. Ca^2+^ transfer from the lysosome to the ER contributes to the replenishing of the ER Ca^2+^ content (63, 64). Preventing this transfer will increase the depletion of the ER Ca^2+^ concentration, and therefore, increasing the Ca^2+^ influx through the STIM–Orai interaction.

Comparable observations in primary T lymphocytes and in 293T cells overexpressing GIMAP5 suggest that GIMAP5-mediated regulation of cellular Ca^2+^ homeostasis involves ubiquitously expressed proteins and processes. It is intriguing that in T lymphocytes, this regulation is linked to cell survival, while cell survival is not affected in 293T, Rat2, or HeLa cells (30). Additional experiments in T lymphocytes and different cell types combined with development of techniques to study lysosomal Ca^2+^ content will be required to understand the contribution of GIMAP5 and lysosomal Ca^2+^ to the cellular Ca^2+^ homeostasis.

The regulation of cellular Ca^2+^ homeostasis by GIMAP5 may be an indirect effect as GIMAP5 does not possess any known Ca^2+^-binding EF-motifs and does not form a Ca^2+^ channel by itself. As GIMAP5 regulates Ca^2+^ responses in HEK293T cells in a manner reminiscent of primary T lymphocytes, it is possible that GIMAP5 interacts with ubiquitously expressed proteins that regulate Ca^2+^ responses in many cell types. Both endogenous and overexpressed GIMAP5v2 regulate cellular Ca^2+^ in a comparable manner, even though the prosurvival function of GIMAP5v2 is restricted to T lymphocytes (30). The presence of GIMAP5v2 increases mitochondrial Ca^2+^ uptake in control T lymphocytes and HEK293T cells expressing GIMAP5v2 (40), without affecting the basal Ca^2+^ content of mitochondria (Figure 6). However, there are certain subtle differences between primary T lymphocytes and HEK293T cells expressing or not GIMAP5v2. In T lymphocytes, disruption of microtubules, but not actin, influenced TG-induced mitochondrial Ca^2+^ uptake, while in HEK293T cells both actin and microtubules appear to be involved (40). Exogenous GIMAP5v2 diminished lysosomal Ca^2+^ stores in HEK293T cells, possibly as a consequence of ER-lysosomal cross-talk (47). However, in contrast to primary T lymphocytes, the influx of Ca^2+^ from the extracellular milieu was still decreased in HEK293T cells expressing GIMAP5v2, but was increased following sequential release from lysosomes and ER (Figure 7C). It is possible that the inability of lysosomes to take up Ca^2+^ in the presence of GIMAP5 disrupts the cross-talk between Ca^2+^ storage organelles, making it difficult for cells to counter balance premature closure by negative feedback of CRAC channels. While the absence of TM domains that anchors GIMAP5v2 to the lysosomal membrane did not affect Ca^2+^ responses of HEK293T cells expressing GIMAP5v2ΔTM, T cells from *Gimap5^lyp/lyp^* rats show decreased Ca^2+^ influx and mitochondrial buffering (39, 40). These observations highlight the relative importance of the various Ca^2+^ stores in different cell types. Despite such differences, HEK293T cells were instrumental in characterizing the association of GIMAP5 with lysosomes and to the microtubule network.

Our results also reveal the complex nature of the interaction between the various subcellular Ca^2+^ stores that are present in a cell, and a role for GIMAP5 in regulating their function in a cell type-specific manner. The Ca^2+^ released from the ER is taken up by ER-associated mitochondria (65). Lysosomes are also found in close association with ER and NAADP-released Ca^2+^ can propagate to the ER and trigger a larger Ca^2+^ release (66). Similarly, bidirectional Ca^2+^ exchange has been shown between ER and lysosomes (67). In yeast, vCLAMP (vacuole and mitochondria patch) are enriched in ions and regulate transport between lysosomes and mitochondria (68). While the presence of lysosomal Ca^2+^ appears to be normal in the physiology of non-T cells as evident from the experiments with HEK293T cells, it is not clear why lysosomal Ca^2+^ is maintained at low levels in T lymphocytes by GIMAP5. It is possible that this reflects the requirement for adequate Ca^2+^ delivery to mitochondria to maintain the energy requirement in T lymphocytes during the initial activation phase. Even though we have observed downregulation of *Gimap5* gene expression following activation, the half-life of the endogenous GIMAP5 protein in T lymphocytes is not known. It is possible that GIMAP5 protein downregulation is also associated with activation and proliferation of T lymphocytes. Thus, turning down the expression of GIMAP5 in activated lymphocytes may permit cell cycle progression (69). Clearly, further studies are needed to elucidate the mechanisms by which GIMAP5 regulate the lysosomal calcium store, and why filling up this store in resting GIMAP5-deficient T lymphocytes compromises survival.

## Materials and Methods

### Animals

*Gimap5^lyp/lyp^* rats and *Gimap5^+/+^* in the ACI.1u background have been described before (30). C57Bl/6 mice were purchased from Charles River (Sherbrooke, QC, Canada). Rats and mice were housed in micro-isolated sterile cages under specific pathogen-free conditions. This study was carried out in accordance with the recommendations of CCPA guidelines. The protocol was approved by the Institutional Université de Sherbrooke, Faculty of Medicine and Health Sciences Animal Ethical Committee. The protocol number is 050-13B.

### Reagents

Tissue culture media, fetal calf serum, CaCl_2_, thapsigargin, anti-FLAG M2 Affinity Gel, and antibodies against α-tubulin, Myc and FLAG, and Carbonyl cyanide 4-(trifluoromethoxy)phenylhydrazone (FCCP) were from Sigma-Aldrich (Oakville, ON, Canada). Gly-Phe-β-naphthylamide (GPN) was obtained from Santa Cruz Biotechnology (Dallas, TX, USA). Nocodazole was obtained from Calbiochem. R73 (anti-rat TCR), H57 (anti-mouse TCR), and IP36 (anti-human TCR) monoclonal antibodies were obtained from Affymatrix-eBiosciences (San Diego, CA, USA). Dynalbeads^®^-M450 tocylactivated beads, Lysotracker Red, and Fura-2 were from Molecular Probes (Life Technologies, Carlsbad, CA, USA). Anti-LAMP2 (H4B4) mAb was obtained from Developmental Studies Hybridoma Bank at the University of Iowa (Iowa, IA, USA). Secondary antibodies for immunoblotting were purchased from Jackson ImmunoResearch Laboratories. Secondary antibodies for fluorescence microscopy were purchased from Thermo Fisher Scientific (Waltham, MA, USA).

Plasmids pmEGFP_α_tubulin_IRES_puro2b (Addgene plasmid # 21042) and pmCherry_α_tubulin_IRES_puro2 (Addgene plasmid # 21043) were kind gifts of Dr. Daniel Gerlich (70). EGFP-IC2-FL (Addgene plasmid # 51409) was a gift from Dr. Trina Schroer (71). LAMP1-mRFP-FLAG (Addgene plasmid # 34611) was a gift from David Sabatini (72). EYFP-KIF5C was a gift from Dr. Anne Stephenson (University of London, London, UK) (73). HA-Rab1a, HA-Rab4, and HA-Rab6a were kind gifts of Dr. Jean-Luc Parent (Université de Sherbrooke) (74). pmRFP-Rab5, pmRFP-Rab7, pDSRed-Rab9, and pDSRed-Rab11 have been already described (75).

### Plasmid Constructs and Cell Lines

Full-length or transmembrane-deleted GIMAP5v2 constructs tagged with an N-terminal Flag-tag, or EGFP tag were subcloned from pCDNA3.1 (30) into pIRES-Puro vector (Figure S1 in Supplementary Material). GIMAP5v1 was cloned from purified rat CD4^+^ T lymphocytes. HEK293T or HeLa cells were transfected using polyethylenimine Max (Polysciences, Inc.) and the stable transfectants were selected using puromycin (2 µg/ml). Protein expression was confirmed by immunofluorescence and by immunoblotting with anti-Flag antibody. The cell lines were cultured in DMEM containing 5% FCS, 1% pen/strep at 37°C in 5% CO_2_ containing atmosphere.

### Negative Selection of Primary CD4^+^ T Lymphocytes

CD4^+^ T lymphocytes were isolated from human peripheral blood and rat and mouse lymph nodes by negative magnetic selection as described previously (37, 39). This study was carried out in accordance with the recommendations of Human Ethics Committee at CRCHUS, Sherbrooke, with written informed consent from all subjects. All subjects gave written informed consent in accordance with the Declaration of Helsinki. The protocol was approved by the “Human Ethics Committee, Centre Hospitalier Université de Sherbrooke (CRCHUS),” Sherbrooke. The protocol number is 14-133.

### RNA Extraction and Quantitative-Reverse Transcription-Polymerase Chain Reaction

Total RNA was extracted using Trizol reagent (Invitrogen, Thermo Fisher Scientific, Waltham, MA, USA) following the manufacturer’s instructions. cDNA was synthesized from 1 µg of RNA using QuantiTect Reverse Transcription Kit (Qiagen, Valencia, CA, USA). Quantitative RT-PCR amplification reactions were carried out in iCycler iQ™ (Bio-Rad, CA) PCR detection systems using iQ™ SYBR^®^ Green Supermix kit (Bio-Rad, Missisauga, ON, Canada). All reactions were run in duplicates along with no template controls for each primer sets. Changes in mRNA expression were calculated using the difference in CT values when compared to housekeeping gene (*Gapdh*) and expressed relative to controls.

### Confocal Microscopy

Stable HEK293T cell lines expressing the GIMAP5 constructs were grown on 15 mm coverslips and were transiently cotransfected with EGFP-α-tubulin, EYFP-KFC5 (Kinesin), or EGFP-IC2-FL (Dynein). Forty-eight hours post-transfection, cells were washed with PBS, fixed with 2% PFA for 13 min, washed, permeabilized with 0.2% saponin for 12 min, washed, and blocked for 20 min with 2% milk in PBS. The slides were incubated with the appropriate primary Ab overnight at 4°C. After washing, the slides were stained with flurochrome-conjugated secondary Abs along with DAPI that stains the nucleus. After the final wash, the slides were mounted with VectaShield (Vector Labs) prior to confocal microscopy. Images were acquired using an Olympus 1 × 81 FV1000 microscope with a 60× oil-immersion objective, and image analysis and Pearson coefficient quantification were done using Fluoview Olympus software 3.1.

For live cell microscopy, HEK293T were grown in coverslips for 48 h before experiments. For purified CD4 T lymphocytes, cells were incubated in RPMI 5% at 37°C for 1 h in previously coated 25 mm coverslips with 100 µg/ml of poly-d-lysine for 2 h at 37°C.

### Calcium Measurements

HEK293T cells grown on 25 mm coverslips for 24–72 h and purified primary T lymphocytes plated on poly-l-lysine coated coverslips were loaded with 1 µM Fura 2-AM for 30 min at 37°C. Coverslips were washed and deesterified for 20 min at room temperature in Ca^2+^ loading buffer (120 mM NaCl, 1 mM Na_2_HPO_4_, 0.5 mM MgCl_2_ 6H_2_O, 5.5 mM glucose, 25 mM HEPES, and 2 mM CaCl_2_, pH 7.3). Coverslips were then placed in a chamber in an Olympus IX71 microscope (Olympus Canada Inc., Markham, ON, Canada) equipped with a Lambda-DG-4 illuminator (Sutter Instrument Company, Novato, CA, USA). Cytosolic Ca^2+^ [Ca^2+^]_c_ measurement was done over an average of 50–70 cells per coverslip per experiment. [Ca^2+^]_c_ was measured at room temperature using two different excitation wavelengths of 340 and 387 nm, and fluorescence emission was monitored at 510 nm through a 415–570 nm dichroic mirror using an Evolve™ EMCCD camera (photometrics, Tucson, AZ, USA). Image digitalization was done using MetaFluor software (Universal Imaging Corporation, Downingtown, PA, USA). In experiments where there was no Ca^2+^ in the extracellular medium, 0.5 mM EGTA was add to the loading buffer instead of 2 mM CaCl_2_ to chelate any extracellular Ca^2+^. Free [Ca^2+^]_i_ was calculated using the Grynkiewicz method (76).

### Western Blot Analysis

Proteins were extracted from cell lysates by homogenizing in RIPA buffer containing protease inhibitors and carried out as described in Ref. (38).

### Statistical Analysis

Statistical analyses were performed using GraphPad Prism 6.0 (GraphPad Software, Inc.; San Diego, CA, USA). Data are presented as mean ± SEM. Statistical significance (considered to be relevant when *p* < 0.05) between two groups was determined by unpaired *t* test.

## Author Contributions

DS and SR planned the experiments, interpreted the data, and wrote the manuscript. FG performed the experiments involving human and murine lymphocytes. SI and CL contributed to the analysis of the data and manuscript revision. GB helped with Fura-2 measurements.

## Conflict of Interest Statement

The authors declare that the research was conducted in the absence of any commercial or financial relationships that could be construed as a potential conflict of interest.

## Supplementary Material

Figure S1 **Gimap5 constructs. GIMAP5 deletions were made in the C-terminal transmembrane domains by PCR cloning**. AIG, AIG1-type guanine nucleotide binding; TM, transmembrane domain. All constructs were tagged at the N-terminus with Flag, RFP, or EGFP tag. Western blot analysis of Flag-tagged GIMAP5 constructs expressed in HEK293T cells.

Figure S2 **GIMAP5v2 does not colocalize with Lysotracker Red**. EGFP-tagged GIMAP5v2 expressing HEK293T cells lines was labeled with Lysotracker Red that accumulates in the lysosomal lumen and analyzed by confocal microscopy. Bar represents 10 µm. Co-localization values are expressed as Pearson’s coefficient. Representative data from four experiments with three to eight cells analyzed per experiment are shown.

Figure S3 **Co-localization of GIMAP5 with tubulin and actin**. Stable transfectants of HEK293T cells expressing full-length and transmembrane-deletion constructs of GIMAP5 tagged with EGFP were transiently transfected with cherry-α-tubulin **(A)** or labeled with phalloidin **(B)** and analyzed by confocal microscopy. Bar represents 10 µm. Colocalization values are expressed as Pearson’s coefficient. Representative data from five to six experiments for **(A)** and from three experiments for **(B)** with four to eight cells analyzed per experiment are shown.

Figure S4 **Co-localization of GIMAP5 with kinesin**. Stable transfectants of HEK293T cells expressing FLAG-tagged GIMAP5 constructs were transiently transfected with EYFP-KIF5C (kinesin) for 48 h and analyzed by confocal microscopy. Bar represents 10 µm. Colocalization values are expressed as Pearson’s coefficient **(B)**. Representative data from three experiments with two to six cells analyzed per experiment are shown.

Figure S5 **Absence of co-localization of GIMAP5 with dynein**. Stable transfectants of HEK293T cells expressing FLAG-tagged GIMAP5 constructs were transiently transfected with EGFP-IC2-FL (dynein) for 48 h and analyzed by confocal microscopy **(A)**. Bar represents 10 µm. Co-localization values are expressed as Pearson’s coefficient **(B)**. Representative data from three experiments with two to five cells analyzed per experiment are shown.

Video S1 **Movement of GIMAP5v2- and LAMP1-expressing vesicles in HEK293T cells**. Stable transfectants of HEK293T cells expressing EGFP-tagged GIMAP5v2 were transiently transfected with cherry-Lamp1. Cells were viewed under 100× epifluorescence microscope for 538 s. In Video S2 in Supplementary Material, cells stable transfected for EGFP-GIMAP5 can be seen and the cell on the left shows the transient transfection for cherry-Lamp1 only.

Video S2 **Movement of GIMAP5v2ΔTM in HEK293T cells**. Stable transfectants of HEK293T cells expressing RFP-tagged GIMAP5v2ΔTM were followed for 5 min. Distribution of RFP-GIMAP5ΔTM seems homogenous and diffuse.

Video S3 **GIMAP5v2-expressing vesicles move along microtubules**. HeLa cells were transiently transfected with vectors containing cherry-α tubulin and EGFP-tagged GIMAP5v2. The vesicles are either filamentous or spherical. The speed of their movement is not uniform. Snapshots of the movement are shown in Figure 4.
