# Supplementary figures and images for: GTPase of the Immune-Associated Nucleotide Protein 5 Regulates the Lysosomal Calcium Compartment in T Lymphocytes
Source: Front Immunol. 2017 Feb 7;8:94. doi: 10.3389/fimmu.2017.00094 (PMC5293772; doi:10.3389/fimmu.2017.00094)

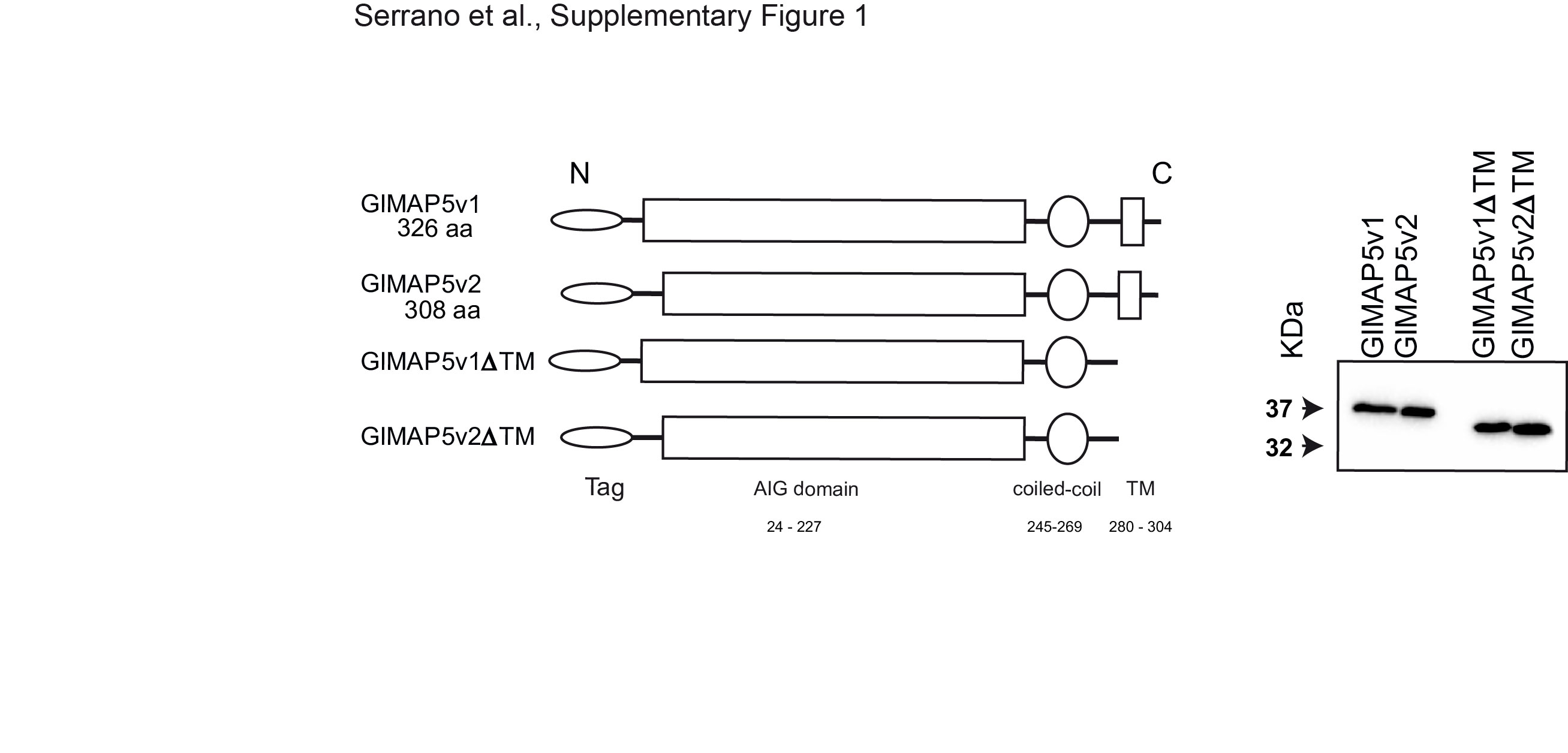

Supplement: Figure S1 — Gimap5 constructs. GIMAP5 deletions were made in the C-terminal transmembrane domains by PCR cloning. AIG, AIG1-type guanine nucleotide binding; TM, transmembrane domain. All constructs were tagged at the N-terminus with Flag, RFP, or EGFP tag. Western blot analysis of Flag-tagged GIMAP5 constructs expressed in HEK293T cells. [file image_1.jpeg]

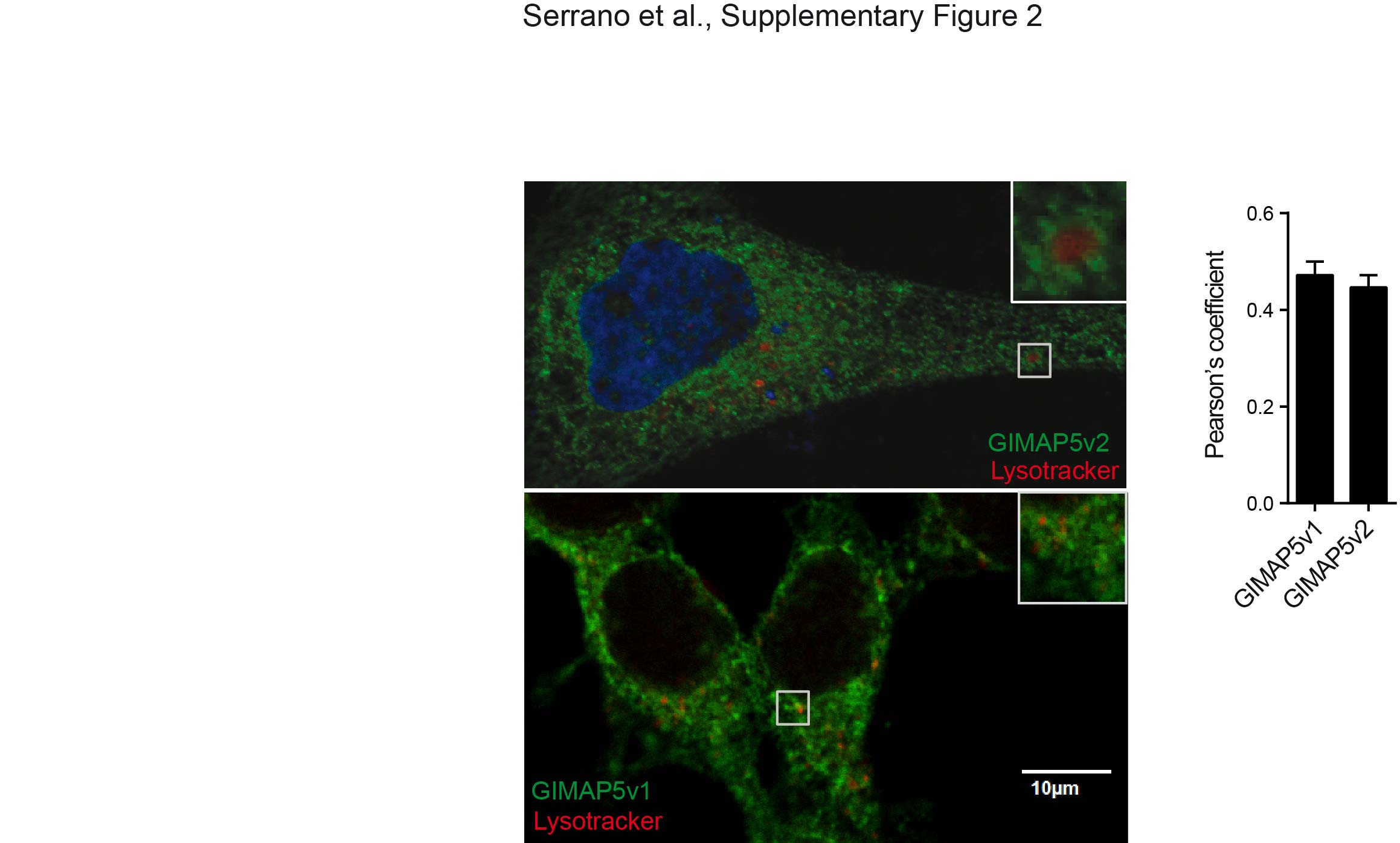

Supplement: Figure S2 — GIMAP5v2 does not colocalize with Lysotracker Red. EGFP-tagged GIMAP5v2 expressing HEK293T cells lines was labeled with Lysotracker Red that accumulates in the lysosomal lumen and analyzed by confocal microscopy. Bar represents 10 µm. Co-localization values are expressed as Pearson’s coefficient. Representative data from four experiments with three to eight cells analyzed per experiment are shown. [file image_2.jpeg]

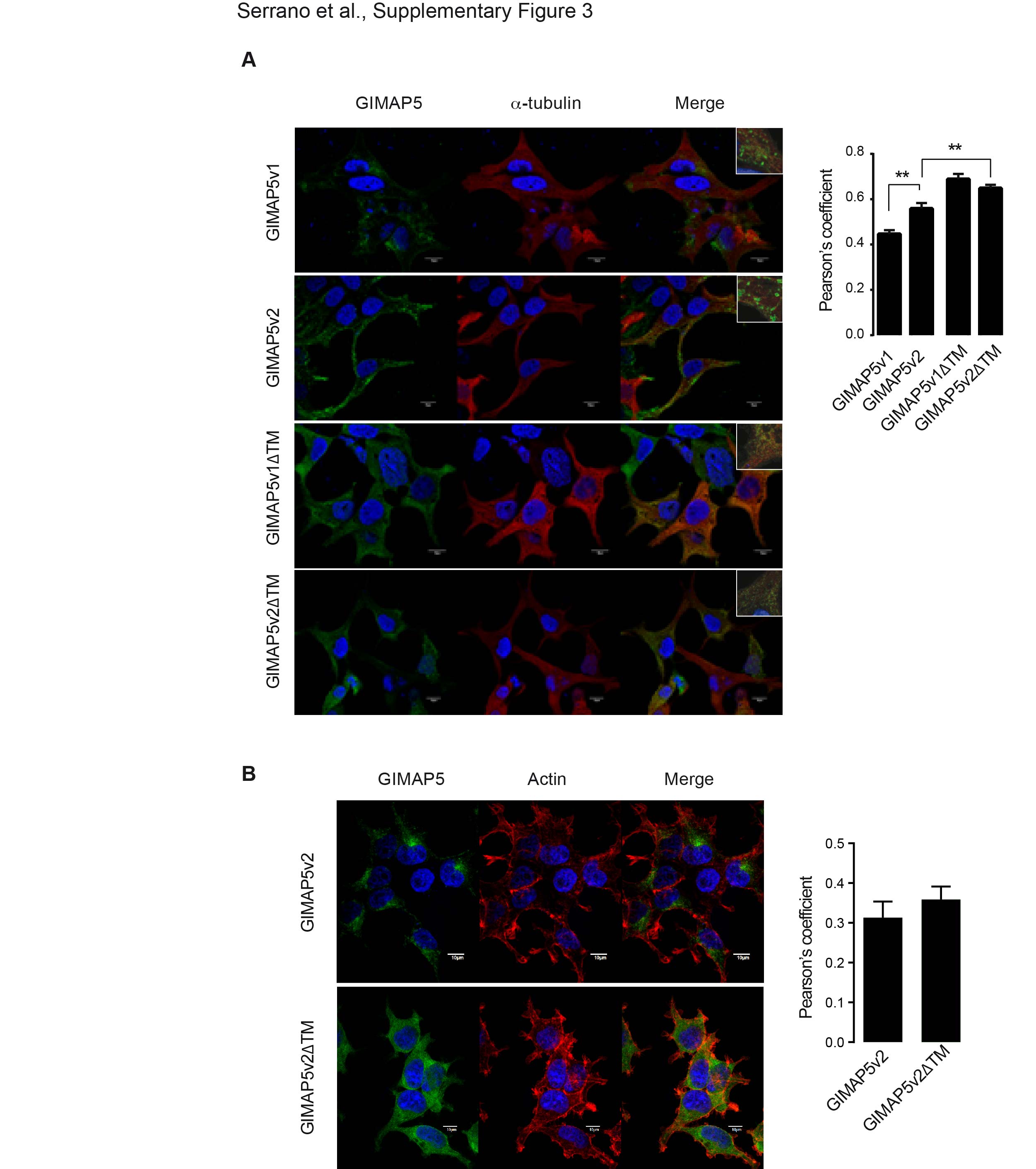

Supplement: Figure S3 — Co-localization of GIMAP5 with tubulin and actin. Stable transfectants of HEK293T cells expressing full-length and transmembrane-deletion constructs of GIMAP5 tagged with EGFP were transiently transfected with cherry-α-tubulin (A) or labeled with phalloidin (B) and analyzed by confocal microscopy. Bar represents 10 µm. Colocalization values are expressed as Pearson’s coefficient. Representative data from five to six experiments for (A) and from three experiments for (B) with four to eight cells analyzed per experiment are shown. [file image_3.jpeg]

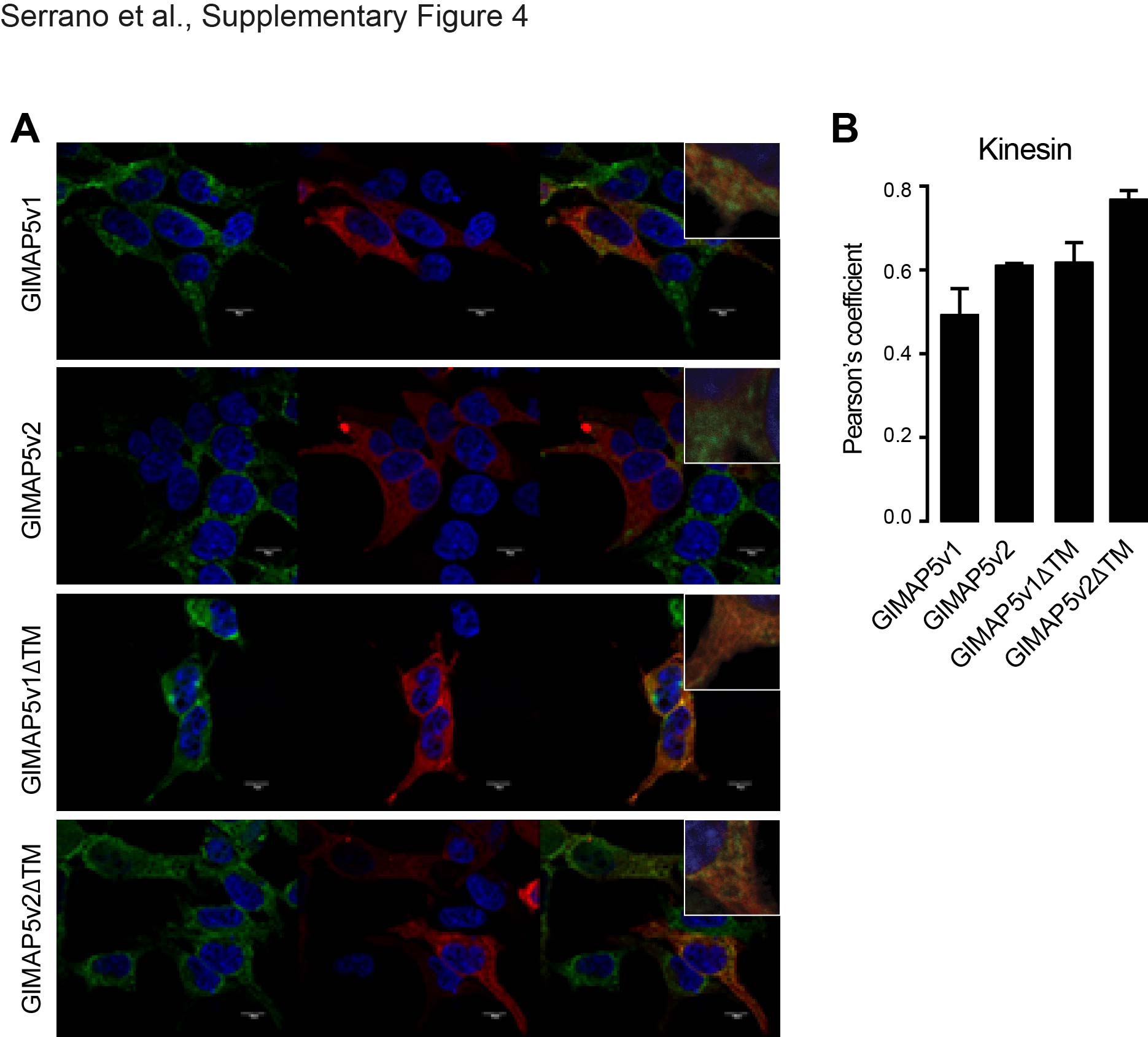

Supplement: Figure S4 — Co-localization of GIMAP5 with kinesin. Stable transfectants of HEK293T cells expressing FLAG-tagged GIMAP5 constructs were transiently transfected with EYFP-KIF5C (kinesin) for 48 h and analyzed by confocal microscopy. Bar represents 10 µm. Colocalization values are expressed as Pearson’s coefficient (B). Representative data from three experiments with two to six cells analyzed per experiment are shown. [file image_4.jpeg]

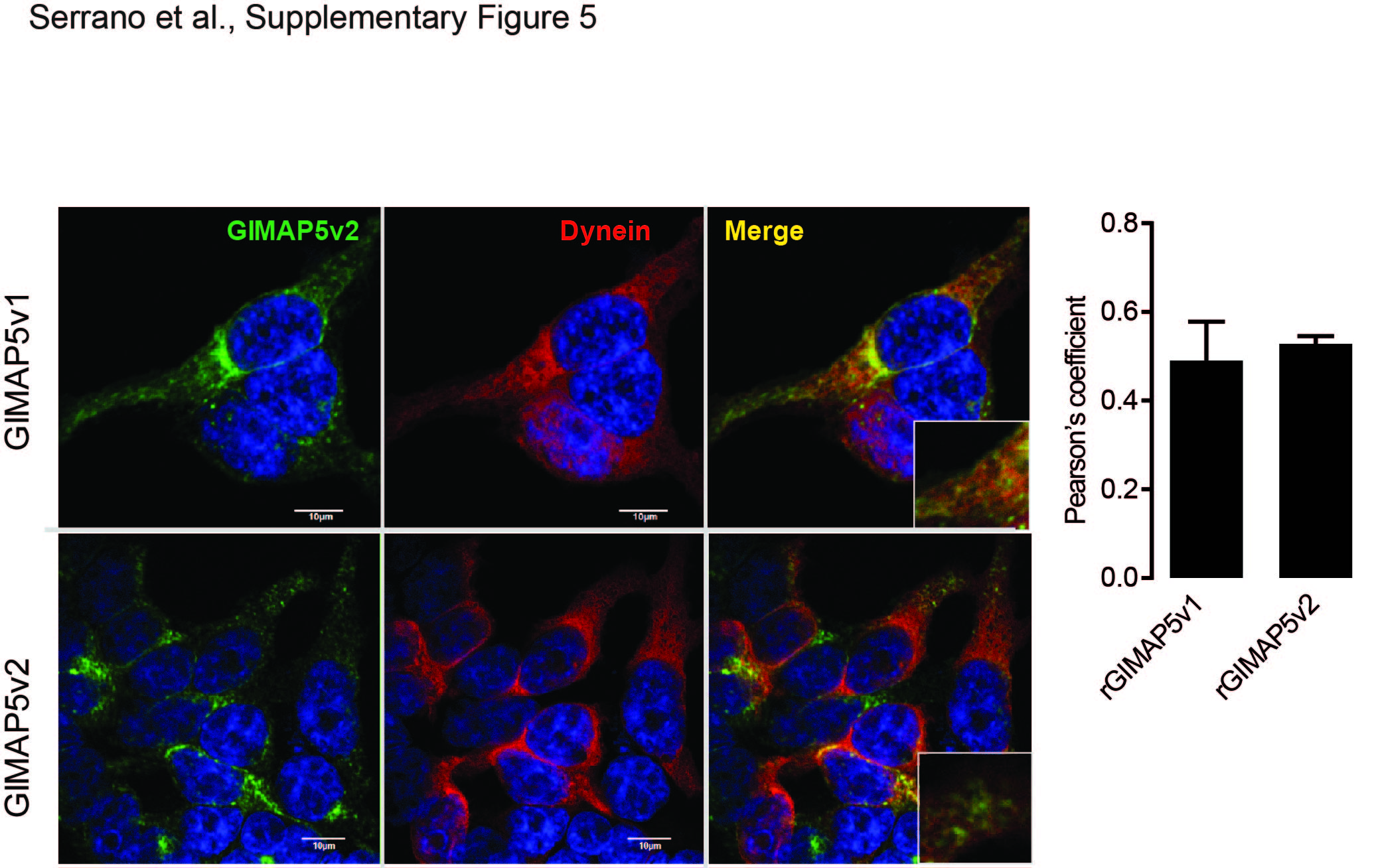

Supplement: Figure S5 — Absence of co-localization of GIMAP5 with dynein. Stable transfectants of HEK293T cells expressing FLAG-tagged GIMAP5 constructs were transiently transfected with EGFP-IC2-FL (dynein) for 48 h and analyzed by confocal microscopy (A). Bar represents 10 µm. Co-localization values are expressed as Pearson’s coefficient (B). Representative data from three experiments with two to five cells analyzed per experiment are shown. [file image_5.jpeg]
